# Anticipatory decision-making for cholera in Malawi

**DOI:** 10.1128/mbio.00529-23

**Published:** 2023-11-14

**Authors:** Antarpreet Jutla, Moiz Usmani, Kyle D. Brumfield, Komalpreet Singh, Fergus McBean, Amy Potter, Angelica Gutierrez, Samuel Gama, Anwar Huq, Rita R. Colwell

**Affiliations:** 1 Department of Environmental Engineering Sciences, GeoHealth and Hydrology Laboratory, University of Florida, Gainesville, Florida, USA; 2 Maryland Pathogen Research Institute, University of Maryland, College Park, Maryland, USA; 3 University of Maryland Institute for Advanced Computer Studies, University of Maryland, College Park, Maryland, USA; 4 Foreign, Commonwealth & Development Office, London, United Kingdom; 5 Office of Water Prediction, National Oceanic and Atmospheric Administration (NOAA), Silver Spring, Maryland, USA; 6 Department of Disaster Management Affairs, Office of the President and Cabinet, Lilongwe, Malawi; University of Tennessee at Knoxville, Knoxville, Tennessee, USA

**Keywords:** cholera, remote sensing, Malawi

## Abstract

Climate change raises an old disease to a new level of public health threat. The causative agent, *Vibrio cholerae*, native to aquatic ecosystems, is influenced by climate and weather processes. The risk of cholera is elevated in vulnerable populations lacking access to safe water and sanitation infrastructure. Predictive intelligence, employing mathematical algorithms that integrate earth observations and heuristics derived from microbiological, sociological, and weather data, can provide anticipatory decision-making capabilities to reduce the burden of cholera and save human lives. An example offered here is the recent outbreak of cholera in Malawi, predicted in advance by such algorithms.

## OPINION/HYPOTHESIS

**Fig 1:**
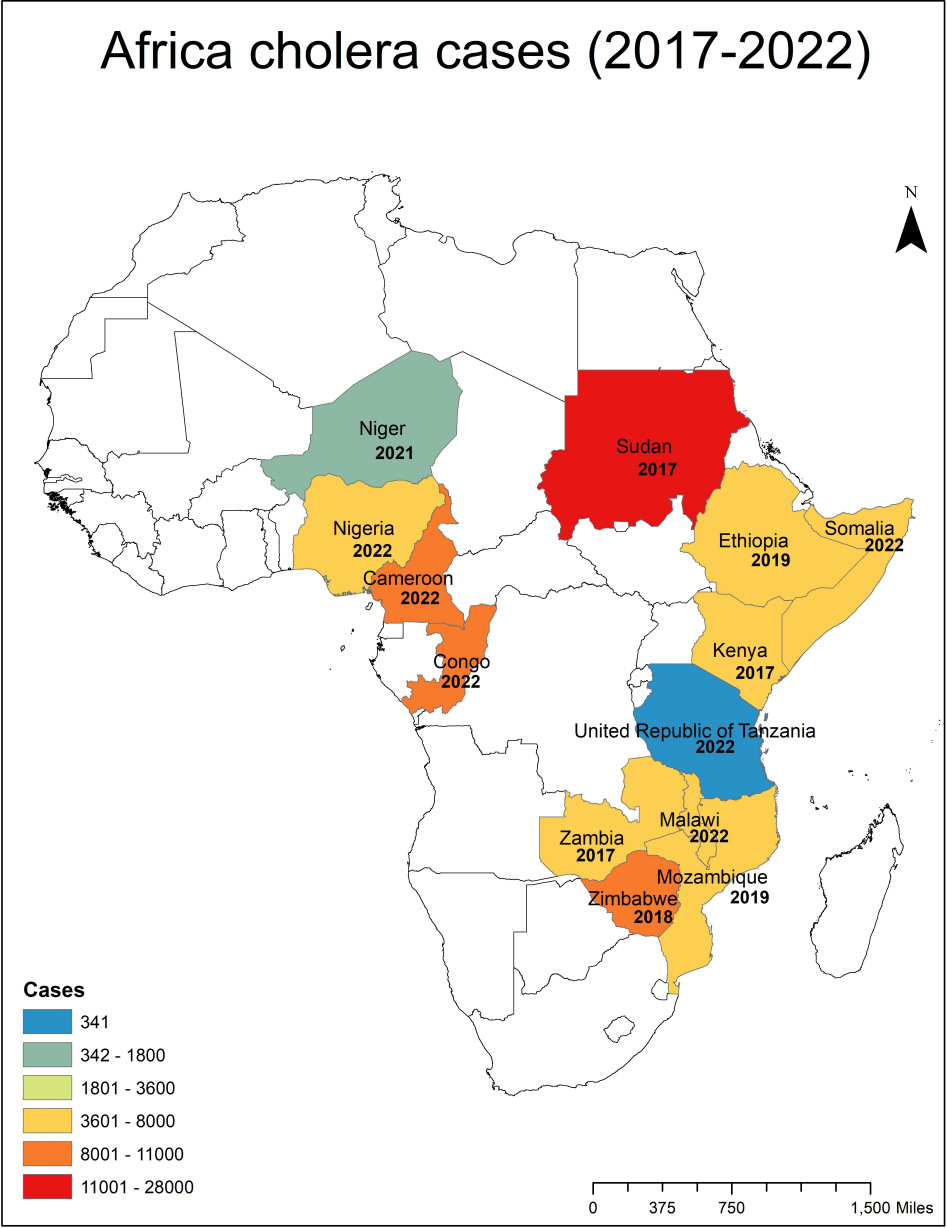
Cholera outbreaks reported in Africa from 2017 to 2022.

Cholera remains a deadly waterborne diarrheal disease and is devastating for populations living in poverty and lacking access to safe water, sanitation, and hygiene (WASH) infrastructure. *Vibrio cholerae*, frequently linked to diarrheal illness and a causative agent of the cholera disease, thrives in regions where environmental, weather/climate, and societal vulnerabilities intersect. The continent of Africa is particularly vulnerable to cholera outbreaks, notably where there is a lack of access to WASH infrastructure and sufficient healthcare facilities. Figure 1 shows major cholera outbreaks occurring across Africa from 2017 to 2022. Apart from African countries, several other countries have reported cholera (1), e.g., Haiti (2010) and more recently in Yemen (2016) (2). Natural (earthquake in Haiti) and anthropogenic (civil unrest in Yemen) disasters have damaged WASH infrastructure (2, 3), resulting in massive cholera outbreaks.

Cholera is preventable by ensuring access to WASH and adequate medical infrastructure. Over the past 50 years, several major discoveries have been made, notably that *V. cholerae* is native to the aquatic environment where it proliferates when conditions for its growth are optimal (4–9). Proliferation of *V. cholerae* and related *Vibrio spp*. in the environment was shown to be driven by environmental factors, namely ambient weather and climatic processes, with coastal waters serving as an ecological niche for several pathogenic *Vibrio spp*., including *Vibrio parahaemolyticus, Vibrio vulnificus,* and *Vibrio cholerae* ([review provided by Brumfield et al. [10]). Another important finding is that *Vibrio spp*. are commensal to copepods, zooplankton comprising a significant component of aquatic fauna that feed on phytoplankton in coastal waters (6, 11). In fact, copepods are a major host of *V. cholerae* (12). A single copepod can harbor up to 10^4^ *V. cholerae* cells (9); hence, ingestion of untreated water containing a small number of copepods can promote disease (13–15), a sufficiently significant activity for the copepod to be concluded a vector (16). Studies by Huq et al. and Colwell et al. (14, 15) demonstrated that employing simple sari-cloth filtration prior to consumption of water effectively removed zooplankton and particulate matter from drinking water and significantly reduced the number of cholera cases in Bangladesh villages. In total, these findings demonstrated vibrios in the environment to be strongly associated with ecological and climate/weather processes (e.g., flooding [17, 18], sea surface temperature [19, 20], zooplankton blooms [12, 14], and salinity [21]] and regional hydrology (e.g., river flows [22], coastal plankton ecology [23], ambient temperature [24], and precipitation [25, 26]).

Previous research demonstrated that cholera outbreaks occur in two modes (27–31): epidemic, which is the sudden occurrence of cholera in a region where societal disturbance results in a lack of access to safe drinking water and appropriate sanitation, and endemic, which is a continuous occurrence of cholera cases in human population with quasi-predictable seasonality. The cholera epidemic mode can evolve to become endemic if WASH access is not ensured. A cholera outbreak requires distinct trigger and transmission mechanisms (29, 30, 32), where the trigger is defined as conditions that initiate an outbreak driven by social and environmental dynamics and transmission as spreading of infection into human communities. While the origins of the cholera trigger have been debated (28, 30), the interaction of humans with an environmental reservoir of *V. cholerae* has been linked with outbreaks of cholera (12, 23, 33, 34).

Given the spatial uncertainty of cholera in vulnerable regions with poor WASH infrastructure, a key challenge is determining when and where to introduce mitigation action to prevent an outbreak. One solution is anticipatory decision-making, a framework that uses predictive intelligence based on knowledge derived from field surveillance and mathematical models (30). A 3-year, near real-time model validation applied in Yemen yielded 72% accuracy in forecasting the risk of the likelihood of cholera (30). It was the first study to highlight the use of environmental, climate, and weather information integrated with microbiological and sociological data to estimate the risk scores for cholera.

A climate-driven, sociological hypothesis states that if a region experiences above-average air temperature, followed by heavy precipitation, and considerable damage to water and sanitation infrastructure, human behavior will change with respect to consumption of water, rendering the region to high risk of cholera (details of the model are provided in previously published studies [23, 28, 31, 35]). The potential of a cholera outbreak will remain low if any of these conditions are not met. A data-driven, score-based mathematical algorithm developed over the past decade provides a reliable lead time of 4 weeks for the risk of cholera (28, 30, 31, 36) (a web hub is currently in beta phase and is available at https://vibrio-prediction-ufl.hub.arcgis.com/). The algorithm provides risk values (high, medium, and low) at 1 km × 1 km pixel scale and employs earth observations, including precipitation, temperature, population density, sociological factors (e.g., access to drinking water and sanitation), and *Vibrio spp*. growth parameters. The output of the algorithm and the validation of the hypothesis have been demonstrated for Zimbabwe (35) and subsequently for Nepal (31) and Haiti (28) and, more recently, for Yemen (30, 36).

**Fig 2:**
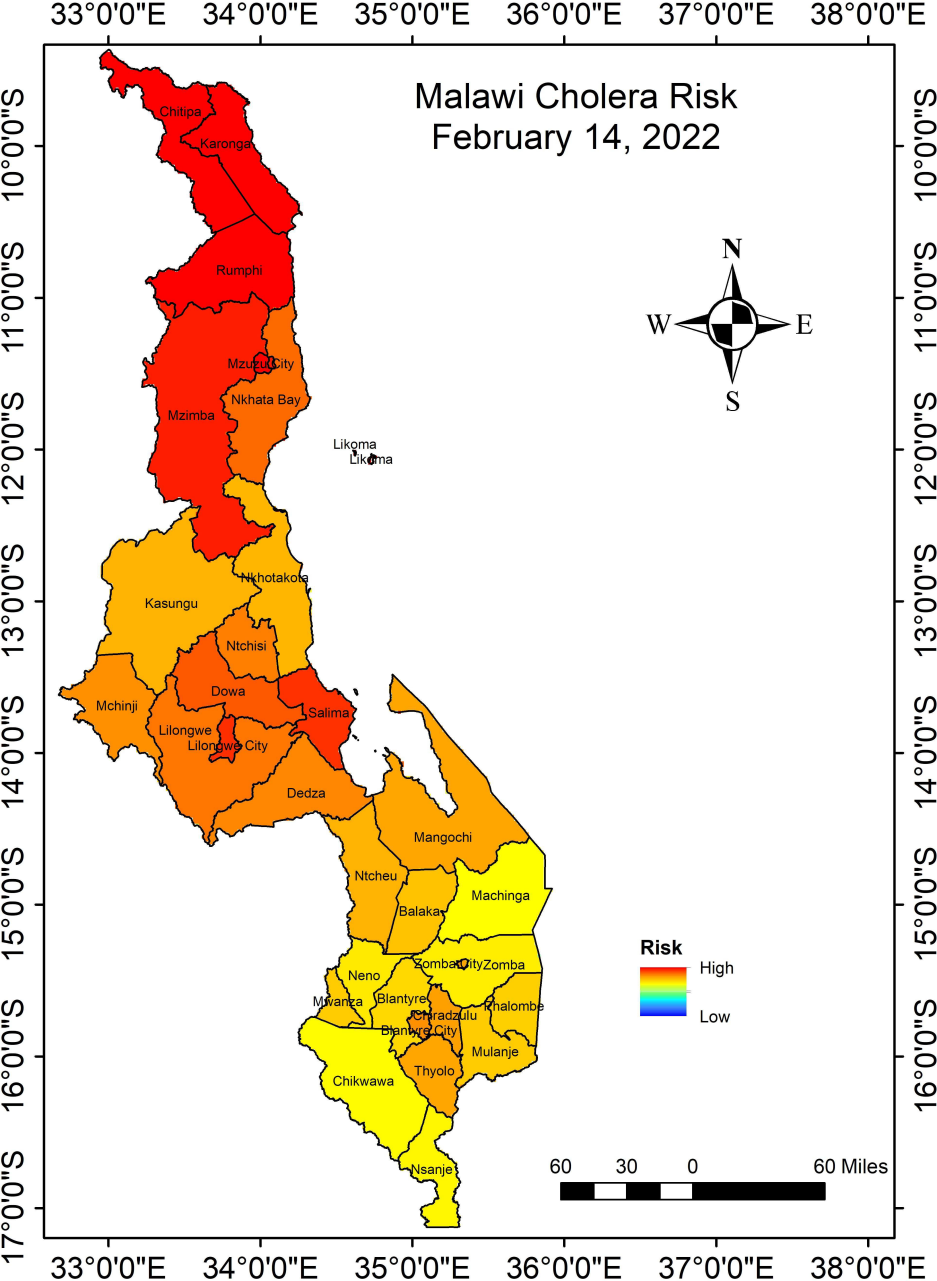
Cholera risk for Malawi 14 February 2022, valid for the following 4 weeks.

**Fig 3:**
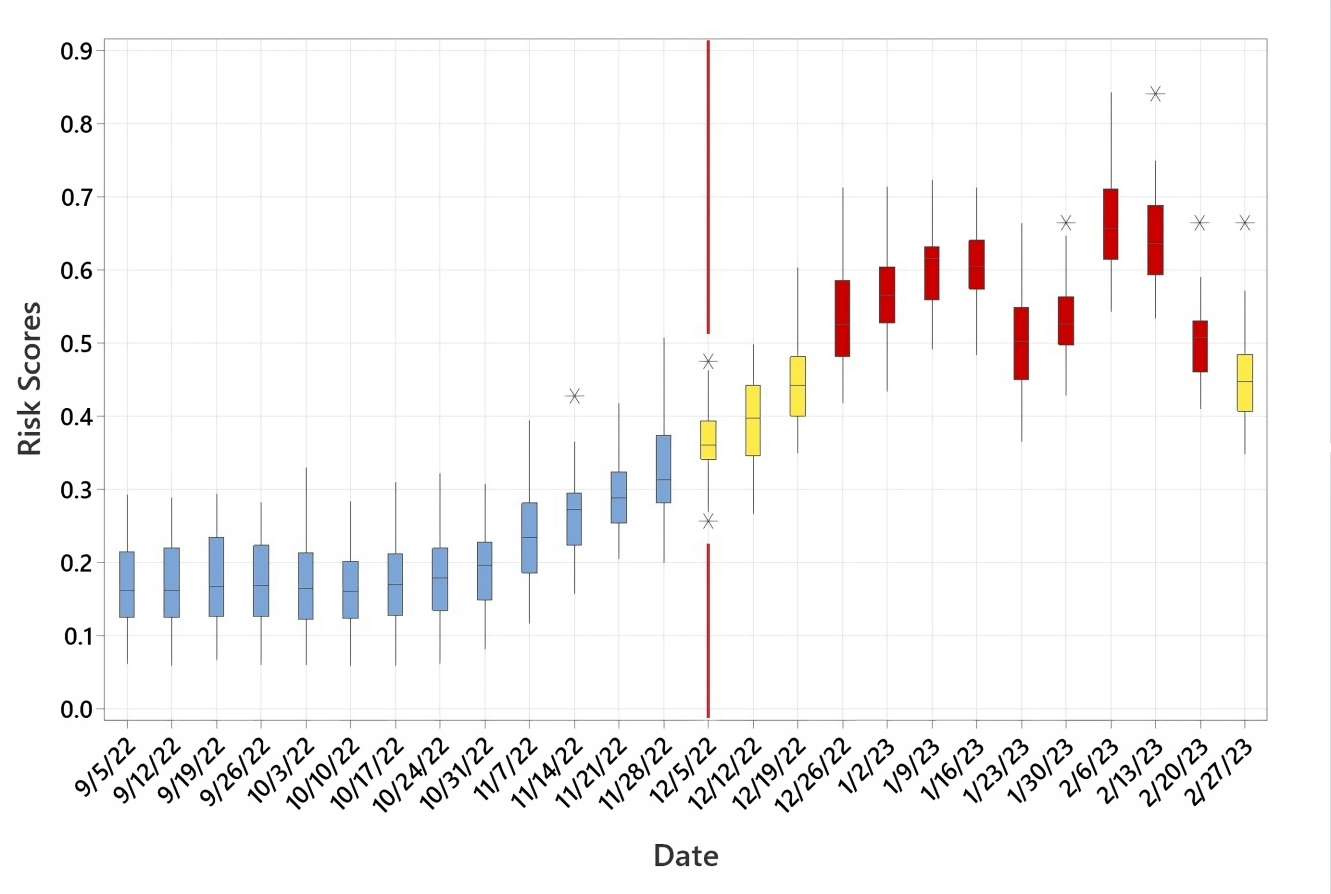
Boxplot for an entire Malawi cholera risk time series (values greater than 0.34 represent medium risk, in yellow color; values greater than 0.50 represent a high risk, in red color, of cholera). Line shows probable time when cholera was acknowledged by the health agencies.

The cholera algorithm, focusing on the trigger mechanism, was implemented in Malawi in February 2022, in the middle of the rainy season. However, the region recorded both anomalous conditions of warm temperatures and high precipitation. Heightened risk of cholera, on a district scale, for the country was predicted (see Fig. 2) with a 4-week lead time. Medium risk, as shown in Fig. 2, indicates that if conditions became amplified (in this case, damage and/or lack of access to WASH infrastructure), the region would experience cholera within 4 weeks of forecast. In fact, the first confirmed case of cholera was reported in Malawi on 2 March 2022 (37), leading the Ministry of Health to declare an outbreak the following day. The cholera cases decreased with the onset of dry months (May to October). Cholera risk, as computed by the algorithm, increased again in October 2022 and peaked in January 2023 (Fig. 3) by which time the outbreak had affected all districts of the country, with case numbers and case fatality surpassing Malawi’s previous worst outbreak 20 years earlier. Cholera risk algorithm produce a time series of risk scores interpreted as a rate of increase (risk value consistently increased over the previous forecasted risk value) (details in references 30, 36). Figure 3 shows the consistent increase in cholera risk from October 2022, hence favored increased odds of cholera.

Geophysical processes have only recently been established for deducing and forecasting the behavior of a pathogen. Therefore, it is crucial to provide a comprehensive, data-driven, and adaptable understanding of an infectious disease that is influenced by weather and climate to achieve reliable decision-making. It is essential to differentiate between reactive and anticipatory decision-making. Most decision-making, with respect to infectious diseases, remain reactive, with intervention and mitigation initiated after an outbreak has begun. Earth observation data, if sociological processes and microbial processes are included, can provide anticipatory decision-making. Frameworks to guide anticipatory decision-making should be developed to support Ministries of Health and other agencies to translate risk data into effective action. This is important in places such as Malawi which are highly vulnerable to increasingly climate-related public health shocks yet with limited resources to respond. For Malawi, anticipatory intervention to limit spread of cholera could have contributed to improving targeted distribution of water safety kits, stockpiling, and ensuring availability of antibiotics, timely vaccination, and education of the local population on handling water drawn from ponds and rivers in conflicted regions. Anticipatory, risk-based intervention in February 2022 could have contributed to preventing or limiting the spread of the initial outbreak that occurred in March 2022, as well as made best use of limited vaccine stocks (38) (given the global shortage) and other interventions by focusing on at risk populations. Thus, country-wide spread of disease that occurred later in 2022 and led to nearly 60,000 cases and over 1,700 deaths could have been prevented. It could also have been helpful to identify when the risk was reducing to inform decisions on when and where to scale down interventions. Internet or data transmission will be effective and helpful in implementing surveillance systems for reporting cholera cases. Table 1 lists some proactive measures that can be employed to prevent major outbreaks of cholera, adapted from reference 39. Reliability of predictive intelligence for infectious diseases generated by mathematical algorithms that integrate earth observations and geophysical processes into disease models is a new field with a powerful future.

**TABLE 1:** Recommended preemptive actions

	Preemptive interventions	Preference	Source
Safe water	Sealed and bottled water	1	(40)
	Water treatment	2	(41, 42)
	Boiling water	3	(43)
Safe defecation	Limit open defecation	1	(44, 45)
	Chemical treatment of fecal matter	2	(46)
	No defecation near/in a water body	3	(47)
Hand wash	Ensuring proper hand washing principles	1	(48, 49)
	Washing hands before and after cooking and eating	2	(47, 49)
	Washing hands when treating sick patients	3	(50)
Eating habits	Thoroughly cooking and preparing food	1	(49)
	Avoiding seafood during disease outbreaks	2	(49)
	Encouraging peeled vegetables and fruits	3	(42, 49)
Oral cholera vaccine	Before exposure (7–10 days before infection)	1	(51)
